# Understanding vessel noise across a network of marine protected areas

**DOI:** 10.1007/s10661-024-12497-2

**Published:** 2024-03-15

**Authors:** Megan F McKenna, Timothy J Rowell, Tetyana Margolina, Simone Baumann-Pickering, Alba Solsona-Berga, Jeffrey D Adams, John Joseph, Ella B Kim, Annebelle CM Kok, Anke Kügler, Marc O Lammers, Karlina Merkens, Lindsey Peavey Reeves, Brandon L Southall, Alison K Stimpert, Jack Barkowski, Michael A Thompson, Sofie Van Parijs, Carrie C Wall, Eden J Zang, Leila T Hatch

**Affiliations:** 1 Cooperative Institute for Research in Environmental Sciences, CU Boulder, National Centers for Environmental Information, National Oceanic and Atmospheric Administration, Boulder, CO USA; 2 Southeast Fisheries Science Center, National Marine Fisheries Service, National Oceanic and Atmospheric Administration, Beaufort, NC USA; 3 Oceanography Department, Naval Postgraduate School, Monterey, CA USA; 4 Scripps Institution of Oceanography, University of California, San Diego, La Jolla, CA USA; 5 Office of Protected Resources, National Marine Fisheries Service, National Oceanic and Atmospheric Administration, Silver Spring, MD USA; 6 Hawaiʻi Institute of Marine Biology, University of Hawaiʻi at Mānoa, Kāneʻohe, HI USA; 7 Lynker in support of Hawaiian Islands Humpback Whale National Marine Sanctuary, National Oceanic and Atmospheric Administration, Kīhei, HI USA; 8 current address: Bioacoustics and Behavioral Ecology Lab, Syracuse University, Syracuse, NY USA; 9 Hawaiian Islands Humpback Whale National Marine Sanctuary, National Oceanic and Atmospheric Administration, Kīhei, HI USA; 10 Saltwater, Inc., Portland, OR in support of NOAA Pacific Islands Fisheries Science Center, Honolulu, HI USA; 11 National Marine Sanctuary Foundation, Silver Spring, MD USA; 12 Office of National Marine Sanctuaries, National Oceanic and Atmospheric Administration, Silver Spring, MD USA; 13 Southall Environmental Associates, Aptos, CA USA; 14 Moss Landing Marine Laboratories, Moss Landing, CA USA; 15 Stellwagen Bank National Marine Sanctuary, National Oceanic and Atmospheric Administration, Scituate, MA USA; 16 Northeast Fisheries Science Center, Woods Hole, MA 02543 USA

**Keywords:** Underwater radiated noise, Marine vessel traffic, Automatic Identification System, National Marine Sanctuary, Sanctuary soundscape project, Soundscape

## Abstract

Protected areas are typically managed as a network of sites exposed to varying anthropogenic conditions. Managing these networks benefits from monitoring of conditions across sites to help prioritize coordinated efforts. Monitoring marine vessel activity and related underwater radiated noise impacts across a network of protected areas, like the U.S. National Marine Sanctuary system, helps managers ensure the quality of habitats used by a wide range of marine species. Here, we use underwater acoustic detections of vessels to quantify different characteristics of vessel noise at 25 locations within eight marine sanctuaries including the Hawaiian Archipelago and the U.S. east and west coasts. Vessel noise metrics, including temporal presence and sound levels, were paired with Automatic Identification System (AIS) vessel tracking data to derive a suite of robust vessel noise indicators for use across the network of marine protected areas. Network-wide comparisons revealed a spectrum of vessel noise conditions that closely matched AIS vessel traffic composition. Shifts in vessel noise were correlated with the decrease in vessel activity early in the COVID-19 pandemic, and vessel speed reduction management initiatives. Improving our understanding of vessel noise conditions in these protected areas can help direct opportunities for reducing vessel noise, such as establishing and maintaining noise-free periods, enhancing port efficiency, engaging with regional and international vessel quieting initiatives, and leveraging co-benefits of management actions for reducing ocean noise.

**Supplementary Information:**

The online version contains supplementary material available at 10.1007/s10661-024-12497-2.

## Introduction

Protected areas often function as a part of a network to achieve overarching goals of biodiversity conservation (Hoffmann, 2022) and climate resilience (Lopazanski et al., 2023) while supporting the human communities reliant on the resources within each protected area. To bolster these goals, efforts are underway to increase the global coverage of protected areas through the “30×30” initiative, an international call for protection of 30% of marine and terrestrial habitats by 2030 (Gurney et al., 2023). Anthropogenic threats to attaining both biodiversity and climate goals vary across protected areas, resulting in a spectrum of environmental conditions and management needs. Without coordinated monitoring of conditions across areas to help prioritize efforts, the effectiveness and equity of the implementation may fall short (Ervin, 2003; Maxwell et al., 2020).

**Fig. 1:**
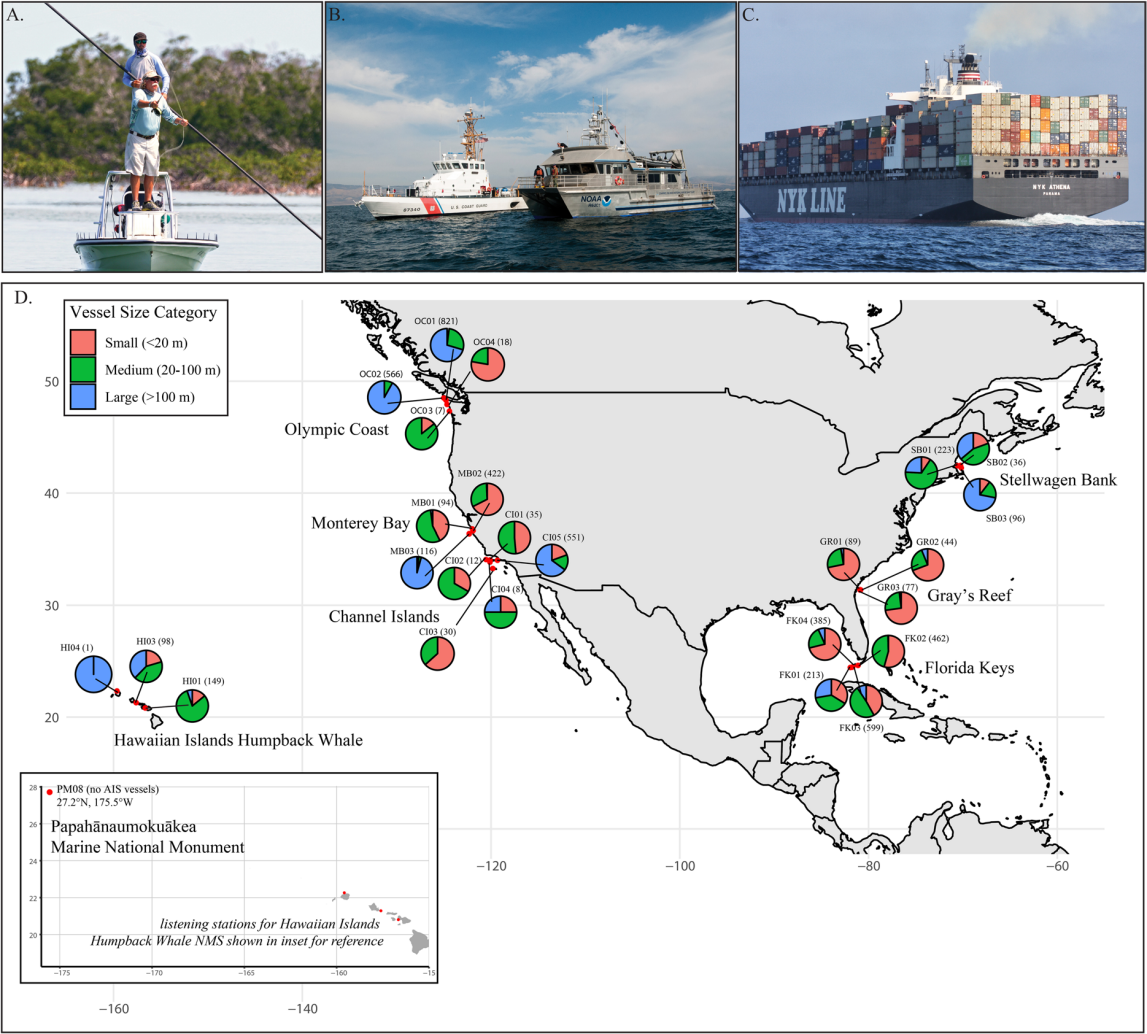
Comparison of AIS vessels operating in U.S. National Marine Sanctuaries (NMS). (A–C) Photographs of different vessel types operating in NMS: (**A**) small recreational fishing vessel in Florida Keys National Marine Sanctuary (credit NOAA), (**B**) research and military vessels operating just north of the Channel Islands National Marine Sanctuary (credit NOAA), (**C**) ocean-going container ships transiting Channel Islands National Marine Sanctuary (credit Cascadia Research Collective). (**D**) Composition of AIS vessel traffic by size categories in a 10 km buffer around each sanctuary listening station. Vessel size categories: small (red) <20 m, medium (green) = 20–100 m, and large (blue) >100 m. A single month in 2019 is shown for each location with count of unique AIS vessels in brackets; month was selected to match availability of acoustic data (Figure S1). AIS data analysis is further described in the Methods section. Due to scale of the map, NMS boundaries are not shown

Monitoring marine vessel traffic across a network of marine protected areas (MPAs) can inform vessel management strategies to reduce a host of threats (e.g., pollutants, greenhouse gas emissions, vessels striking whales, noise) in protected areas and beyond. Marine vessel operations in MPAs support diverse human needs and activities (Fig. 1A–C). Ocean-going container ships and tankers transit coastal waters delivering goods to nearby ports. Military, search and rescue, and law enforcement vessels support the health and safety of mariners, uphold coastal regulations, and conduct training and testing activities. Tug-tows, service vessels, and specialized vessel types assist offshore construction and operations. Research vessels carry out monitoring activities and support scientific inquiry. Vessels such as day cruisers, charter vessels, passenger vessels, trawlers, dredgers, and seiners transport wildlife viewers and fishing operations. Many of these vessel-dependent activities rely on a healthy marine ecosystem for both economic and well-being benefits, yet vessel presence can alter ecological conditions. Threats from vessels are well-documented with consequences to marine species and ecosystems, including the introduction of invasive species (Iacarella et al., 2020), behavioral and sensory disturbances (Erbe et al., 2019; Weilgart, 2018), fatal collisions with wildlife (Schoeman et al., 2020), oil spills (Dalton & Jin, 2010), and air-quality concerns (Viana et al., 2014). Vessels operating in protected areas are sometimes allowed to extract marine resources, such as fish (Rowlands et al., 2019). Capturing the variety in vessel operations, including types, movement patterns, and volume, across a network of MPAs can help quantify the relative magnitude of threats and prioritize and coordinate management needs.

Technologies to monitor marine vessels continue to advance. The various technologies work to ensure vessels of interest are captured, the spatial and temporal coverage is sufficient for management objectives, and derived metrics are sensitive to changes in activity and related impacts (de Oliveira et al., 2019; Rowlands et al., 2019). Depending on the monitoring objective, each technology has strengths and limitations. Visual surveys within a defined region provide a census of vessels, yet on a limited temporal and spatial scale (Hermannsen et al., 2019). Vessel monitoring systems (VMS), which include a transmitter-receiver system, provide information on a particular pre-defined sector, typically fishing vessels targeting federally managed fish stocks (Birchenough et al., 2021). Reporting of vessel behavior at a fine scale using radar can help resource managers target enforcement efforts and understand human use patterns within coastal marine protected areas (Cope et al., 2020, 2022). Satellite imagery has been used to detect vessels (Müller et al., 2013; Paolo et al., 2024), expanding monitoring further offshore. The International Maritime Organization (IMO) developed a technical standard for tracking vessels through transmission of very-high-frequency (VHF) radio signals, called the Automatic Identification System (AIS), which can be monitored using satellites or shore-based systems. AIS is a mandatory vessel communication and navigational safety system for commercial vessels greater than 300 gross tonnage that was adopted by the IMO in 2000 for use in collision avoidance, coastal surveillance, and traffic management. Although AIS was not designed with research or conservation planning in mind (Robards et al., 2016), many studies have demonstrated the multifaceted benefits of this monitoring data (Welch et al., 2022). Listening for underwater vessel noise using passive acoustic monitoring systems provides information on vessel presence (Haver et al., 2023; Kline et al., 2020), specific noise output (ZoBell et al., 2021, 2023), and cumulative noise added to the marine environment (Haver et al., 2021). The spatial scale of detecting vessel noise depends on sound propagation conditions in the local environment, while the temporal scale varies by instrumentation limitations and project goals (e.g. length of instrument deployments, duty cycling, or seasonality of data collection). Integrating several of these vessel monitoring technologies can broaden the diversity of vessel types monitored, as well as the spatial extent and temporal coverage of the data (O’Hara et al., 2023). These integrated methods can support monitoring of different vessel-related threats to protected areas, such as underwater radiated noise.

Motorized marine vessels produce underwater radiated noise that propagates into the surrounding water and degrades the quality of the habitat for species dependent on hearing sounds for communication and other life functions (Duarte et al., 2021; Erbe et al., 2019). Efforts to reduce noise impacts from vessels are emerging with solutions ranging from vessel slowdown initiatives to engineering solutions and policy options (Chou et al., 2021). Leveraging networks of MPAs to reduce noise impacts holds benefits for areas beyond the protected area borders as well as achieving effectiveness and efficiency at local to network-wide scales.

Here, we explore vessel noise within the U.S. National Marine Sanctuary (NMS) system, a network of underwater parks encompassing more than 1,600,000 square km of marine and Great Lakes waters (Murley et al., 2021). The network includes 15 national marine sanctuaries and Papahānaumokuākea and Remote Islands marine national monuments, with additional areas currently under review (https://sanctuaries.noaa.gov/). Each sanctuary is uniquely embedded within national, regional, and local vessel operations, yet we can leverage its geographic scope to assess cross-cutting needs for multiple sanctuaries (Massaua, & Alexander, 2021). Sanctuary-wide metrics for vessel noise as well as other stressors are needed to compare and categorize conditions across the system and monitor changes related to both internal factors (e.g., management actions) and external drivers (e.g., global economics).

We used underwater acoustic detection of vessels to quantify and categorize network-wide patterns of radiated vessel noise by separating the soundscape into vessel noise and non-vessel noise periods. We integrated acoustic metrics with AIS vessel monitoring technology to further interpret differences in vessel noise conditions. We applied standardized system-wide vessel noise metrics to explore finer temporal (daily and seasonal) trends and evaluate sensitivity of the metrics by examining shifts related to management actions (mandatory vessel slowdowns) and external drivers (COVID-19 pandemic). Collectively, this study presents an extensive effort to understand vessel noise across a diverse network of MPAs. Combining this knowledge with an understanding of vulnerable resources and community needs can help inform efficient and effective approaches to reducing vessel noise within the U.S. National Marine Sanctuary System, and example MPA network.

## Methods

### Sanctuary soundscape monitoring project

Data analyzed in this project were part of a multi-year effort (2018–2022) to monitor underwater sound within the U.S. National Marine Sanctuary (NSM) system. Beginning in 2018, the U.S. National Oceanic and Atmospheric Administration (NOAA) and the U.S. Navy worked with dozens of scientific partners to study sound within seven national marine sanctuaries and one marine national monument, including monitoring locations off the east coast of the U.S. (Stellwagen Bank, Gray’s Reef, and Florida Keys National Marine Sanctuaries), the west coast of the U.S. (Olympic Coast, Monterey Bay, and Channel Islands National Marine Sanctuaries), and the central Pacific region (Hawaiian Islands Humpback Whale National Marine Sanctuary and Papahānaumokuākea Marine National Monument) (Fig. 1D). As the first coordinated monitoring effort of its kind for the NMS system, the Sanctuary Soundscape Monitoring (known as SanctSound) project was designed to provide standardized acoustic data collection and analysis products to document sound levels and types of sound sources occurring within these protected areas as well as quantify potential impacts of noise to the areas’ marine taxa and habitats.

A total of 28 locations, referred to as listening stations, were selected to represent a variety of habitats and human use patterns within the NMS system. Collectively, the SanctSound project used a variety of acoustic data processing techniques (e.g., sound pressure levels in designated frequency bands, source-specific automated detectors, manual audits), incorporated complementary data sources (e.g., from gliders, ship traffic data, weather stations), and estimated sound source detection ranges using sound propagation models (McKenna et al., 2021). Project data products can be visualized, explored, and downloaded via the project data portal (https://sanctsound.portal.axds.co/) and raw audio files are available through the National Center for Environmental Information (NOAA Office of National Marine Sanctuaries and U.S Navy, 2020).

### Listening for vessels

Twenty-five of the 28 listening stations in the SanctSound project were analyzed to understand patterns in vessel noise conditions using multiple metrics (see the “Vessel noise metrics” section). Data from these 25 locations varied based on data availability (e.g., gaps in data related to delays in deployment/recovery, seasonal recording schedules, data quality concerns) (Fig. S1). One month of data in 2019 with sufficient data (>95% of days in the month) was used to compare vessel noise conditions across the 25 listening stations; when possible data collected in either April or May were analyzed. At 4 of these 25 locations (SB03, CI05, FK03, MB02), daily patterns in vessel noise were analyzed (Fig. S1). Only 2 listening stations (SB03, GR01) with complete data for all months in 2019 were used to evaluate seasonal patterns which included a mandated vessel speed reduction period at one of the locations (SB03). Ten listening stations with data in April of 2019 and 2020 were used to understand changes in vessel noise related to early COVID-19 shutdowns. Previous studies have used both 63-Hz and 125-Hz one-third octave bands as representative of noise from marine vessel traffic (European Commission, 2010; Haver et al., 2021). This study summarized sound pressure levels in the 125 Hz one-third octave band, which is representative of a wide range of vessel sizes.

Acoustic data were collected using SoundTraps (ST300, ST500, and ST600), which are compact, self-contained underwater sound recorders developed by Ocean Instruments Inc. (http://www.oceaninstruments.co.nz). Across the SanctSound sensor network, instruments recorded continuously at a sampling rate of 48 kHz or 96 kHz. Hourly power spectral density (PSD) levels were calculated as the median of mean-square pressure amplitude (µPa^2^) with a resolution of 1 Hz/1 second from 20 to 24,000 Hz over no less than 1800 s in each hour and converted to decibels (dB re 1 µPa^2^/Hz). One-third octave band (also commonly referred to as decidecade bands) sound pressure levels were calculated by integration of PSD levels with a 1 Hz/1 s resolution over each one-third octave frequency band with nominal center frequencies ranging from 125 to 20,000 Hz and summarized as medians per hour. Bands below a nominal center frequency of 125 Hz were not included in our analysis. For some listening stations, minute resolution band levels were also calculated from the PSD levels as the median of mean-square pressure amplitude (µPa^2^) with a resolution of 1 Hz/1 s and summarized into one-third octave bands. All calculations were performed using Matlab^TM^ (Mathworks, Natick, MA). Code for calculating calibrated sound pressure levels from audio recordings is available on GitHub (https://github.com/MarineBioAcousticsRC/Triton.git), specifically the Soundscape Metrics package.

### Vessel noise metrics

To divide the soundscape into vessel noise and non-vessel noise periods, we used a previously developed vessel noise detector, Triton Ship-Detector (Solsona-Berga et al., 2020) available on GitHub (https://github.com/MarineBioAcousticsRC/Triton/tree/master/Remoras/Ship-Detector). Settings for the detector varied by sanctuary to account for inherent difference in the soundscapes, including the background sound contributions (Table S1). In brief, long-term spectral averages (LTSAs) were created for each site with a 5-s 48-Hz resolution and vessel events were automatically identified from these LTSAs. Individual site settings were tested using the Interactive Detector, and the analyst selected the final settings to run the Batch Detector. The LTSAs were analyzed in blocks of data with buffers to be able to classify events that are at the edge of the block of data, where calibrated PSD estimates were averaged in three defined frequency bands: low, medium, and high. If the three averaged PSDs met specific criteria, start and end times of the events were considered preliminary vessel event detections and stored. The detection criteria included the following: (1) Amplitude was above a user-defined time-dependent amplitude threshold which was computed using a histogram method of the averaged PSDs at a specific site and recording period. (2) Vessel noise detections were distinguished from marine mammal echolocation click events when noise detection duration was above a user-defined time in the three bands. Specifically, the duration in the high band must have been shorter than the medium band, or the detection duration above the threshold in the low and medium bands must have been longer than the user-specified time, and the duration in the medium band must have been shorter than the low band. (3) Vessel noise detections were distinguished from weather events when averaged received levels of the event in the low band were above a user-specified percentage of the background sound in the window. After the Batch Detector was run, a trained analyst visually and aurally reviewed results and corrected labeled events to confirm either a non-vessel source or vessel event. Trained analysts used example sound clips of known vessel noise to help evaluate the detections.

Metrics extracted from the verified vessel detections provided insight on how often vessel noise dominated the soundscape (referred to as vessel noise dominance metrics in this study) (Table 1 and Table S2). The specific vessel noise dominance metrics extracted from vessel noise detections included the following: percentage of time vessel noise was detected (within day, hour, or month), durations of vessel noise detections, and counts of vessel noise detections (within day, hour, or month) (Table 1). These vessel noise metrics represented presence of vessel noise in the soundscape, but did not distinguish between overlapping noise from multiple vessels.

**Table 1:** Summary of vessel noise metrics

Metric	Description	Figure	Interpretation (*informed by previous reports and studies*^12345^)
Proportion of AIS traffic small-sized vessels (<20 m)	Total of all unique small vessels equipped with AIS within a 10 km buffer around a listening station divided by total vessels equipped with AIS. Metric is calculated for the entire month.	1	Higher values indicate vessel traffic and associated noise are from local sectors (e.g., recreation, Fig. 1A), often with high-frequency noise signatures and less predictable routing
Proportion of AIS traffic medium-sized vessels (20–100 m)	Total of all unique medium vessels equipped with AIS within a 10 km buffer around a listening station divided by total vessels equipped with AIS. Metric is calculated for the entire month.	1	Higher values indicate vessel traffic and associated noise are from local and regional sectors (e.g., research and military, Fig. 1B) often with mid-frequency noise signatures and a mix of routes
Proportion of AIS traffic large-sized vessels (>100 m)	Total of all unique large vessels equipped with AIS within a 10 km buffer around a listening station divided by total vessels equipped with AIS. Metric is calculated for the entire month.	1,2	Higher values indicate vessel traffic and associated noise are from regional to international sectors (e.g., international commerce, Fig. 1C) often with lower-frequency noise signatures and more predictable routing
Percent of hours vessel noise dominant (hourly resolution)	Total number of hours with at least one acoustic vessel detected in a month divided by total hours sampled in that month.	2	Higher values indicate that vessel noise is a dominant component of the soundscape.
Low-frequency vessel noise exceedance (hourly resolution)	Difference in average hourly sound level when vessel is acoustically detected minus average hourly sound level when vessel is not acoustically detected. Metric is calculated for each month. Sound levels in the 125 Hz one-third octave band (112–141 Hz) are used.	2	Higher values indicate that vessels add more noise to the environment because vessels have higher source levels and/or are transiting nearby and sound levels are low when vessels are not present.
Vessel noise exceedance and dominance category	A two-dimensional framework to compare listening stations within and across sanctuaries based on both the amount of time that vessel noise was present in the soundscape (dominance) and the level of noise added by vessels (exceedance).	2	Listening stations that fall in the high-dominance, high-exceedance category indicate a vessel’s contribution to the soundscape is both continuous and loud.
Duration of vessel detection periods	Total minutes in each hour with vessel noise dominate averaged for each hour of the day for a given month. Standard error calculated for each hour within the month.	4	Higher values indicate shorter vessel noise-free periods in a given hour of the day. Larger variations within an hour indicate vessel activity related to day of the week, weather, or time of the month.
Low-frequency vessel noise exceedance (minute resolution)	Difference in median and max sound level during vessel detection period from nearest in time average sound level when vessel not detected.	3, 5	Higher values indicate that vessels add noise above other sounds in the soundscape when present.
Low-frequency sound level in 125 Hz one-third octave band	Power spectral density (PSD) levels per hour calculated as the median of mean-square pressure amplitude (µPa^2^) with a resolution of 1 Hz/1 s from 20 to 24,000 Hz over no less than 1800 s in each hour and converted to decibels (dB re 1 µPa^2^/Hz). One-third octave band sound pressure levels (TOLs) were calculated by integration of PSD levels with a 1 Hz/1 s resolution over each one-third octave band. Metric is for the 125 Hz (112–141 Hz) one-third octave band.	5, 6	If nearby large vessel traffic is high, higher levels indicate contributions of low-frequency energy from nearby large vessels If nearby large vessel traffic is low, higher levels indicate contributions from other low-frequency sources (e.g., fish)
Low-frequency vessel noise exceedance (minute resolution) AIS vessel nearby	Difference in median and max sound level during acoustic vessel detection period with at least one known AIS vessel transiting within 10 km of the listening station from nearest in time average sound level when vessel not detected.	5	Higher values indicate that AIS vessels add noise to the soundscape when present.

^1^Kipple, B. M., & Gabriele, C. M. (2003). *Glacier Bay Underwater Noise-August 2000 through August 2002*. Naval SurfaceWarfare Center-Carderock Division, Technical Report, NSWCCD-71-TR-2004/521.

^2^Hatch, L., et al. (2008). Characterizing the relative contributions of large vessels to total ocean noise fields: a case study using the Gerry E. Studds Stellwagen Bank National Marine Sanctuary. *Environmental Management*, *42*, 735–752. 10.1007/s00267-008-9169-4

^3^Parsons, et al. (2021). A review and meta-analysis of underwater noise radiated by small (< 25 m length) vessels. *Journal of Marine Science and Engineering*, *9*(8), 827. 10.3390/jmse9080827

^4^McIntyre, et al. (2021). Influence of propellers and operating conditions on underwater radiated noise from coastal ferry vessels. *Ocean Engineering, 232*, 109075. 10.1016/j.oceaneng.2021.109075

^5^Cauchy, P et al. (2023). Measurement of Vessel Underwater Acoustic Signature–Repeatability Assessed on the Mars Database. *Canadian Acoustics, 51*(3), 234–235.

Other sources of sound contribute to low-frequency measured sound levels (e.g., fish), creating temporally varying soundscapes to which vessel noise is added. To quantify variation in noise added from vessels (hearby referred to as noise exceedance in this study), we compared the 125 Hz one-third octave sound levels (hourly and minute resolution) when vessels were acoustically detected (vessel noise detections) to the closest periods when vessels were not detected. For each vessel noise detection, 1-min sound levels were summarized as median and maximum sound levels (Table 1).

All processing was conducted in R statistical software (version 2022.02.1 Build 461) and code is available on GitHub (https://github.com/mfmckenna/MM_SanctSound_VesselNoise).

### Vessel noise categories

Vessel noise metrics for a month of data in 2019 from 24 listening stations (one station did not have vessel noise detections, so categories only applied to 24 of the stations) were used to compare conditions across distinct compositional, spatial, and temporal vessel traffic conditions (Fig. 1D). We created a two-dimensional framework to compare listening stations within and across sanctuaries based on both the amount of time that vessel noise was present in the soundscape (dominance) and the level of noise added by vessels (exceedance). To help interpret patterns, the framework can be thought of in four categories in four quadrants of the graph: high-dominance, high-exceedance (upper right); high-dominance, low-exceedance (upper left); low-dominance, high-exceedance (lower right); low-dominance, low-exceedance (lower left. Vessel traffic information from AIS data within 10 km buffer of station (% large vessels and proximity to commercial shipping lanes) helped with interpretation of the categories (Fig. 1D, Table 1).

### Detecting change in vessel noise

In addition to system-wide comparisons of vessel noise categories, the derived vessel noise metrics (Table 1) were also used to examine finer temporal patterns and sensitivity of these metrics to known changes in vessel activity. Two listening stations with complete data for all months in 2019 were used to evaluate seasonal patterns in vessel noise dominance and exceedance. To understand temporal variation, seasonal and daily, vessel noise metrics and AIS vessel counts were compared across months and within hours of a day. These analyses provided insight on the use of vessel noise dominance and exceedance metrics, which may help inform management strategies in MPAs (e.g., when and where to focus management efforts).

Understanding the effectiveness of vessel management action(s) is another priority in protected area management. Measures of individual ships provide valuable insight on per-ship noise reductions (ZoBell et al., 2021) and, the NMS system managers also need to understand the noise reduction benefit at specific locations, sensitive habitats, or for species of interest (Burnham et al., 2023). Within our study period, a mandatory vessel slowdown at Stellwagen Bank NMS occurred and provided an opportunity to understand how the vessel noise metrics calculated in this study changed with this specific management action. The mandatory vessel slowdown occurred in March–April 2019 and required vessels 20 m or longer to slow down to 10 knots or less to reduce the collision risk with endangered North Atlantic Right Whales (*Eubalaena glacialis*), with a possible co-benefit of reducing ocean noise (Findlay et al., 2023). We evaluated noise reduction benefits at one listening station in Stellwagen Bank NMS.

Detecting changes in conditions related to unexpected shifts in vessel activity within the early COVID-19 pandemic offered an opportunity to understand sensitivity of the vessel noise metrics across a variety of vessel traffic conditions. Nine listening stations with data from April of 2019 and 2020 were used to examine if and how vessel noise metrics changed as a result of shifts in marine vessel traffic during the early COVID-19 shutdowns.

### Vessel traffic composition

The presence and composition of marine vessels equipped with AIS transponders were summarized for a 10 km buffer around each listening station; the 10 km buffer was intended to represent nearby AIS vessel use. AIS vessels periodically transmit information on vessel position and speed, as well as static vessel parameters such as name, unique identifier, type, and length. Given the relationship between vessel size and both vessel use patterns and acoustic characteristics (McKenna et al., 2012, 2013), vessel size categories for the AIS vessels were created based on vessel length: small (<20 m), medium (20–100 m), large (>100 m), or unknown when data on length was unavailable.

Metrics extracted from the AIS data within the 10 km buffer around a listening station included the proportion of vessels in each size category, the count of unique AIS vessels present for each size category, and the total operational hours of all AIS vessel tracks (Table 1). These metrics were calculated as daily summaries and used to calculate monthly averages and standard deviations.

## Results

Data from 25 listening stations across a network of marine sanctuaries off the U.S. east and west coasts and Hawaiian Islands (Fig. 1D, Fig. S1) revealed distinct patterns in vessel noise (dominance and exceedance), temporal patterns in conditions, and changes related to external drivers in vessel activity. AIS data, summarized in a 10 km buffer around each listening station, was available for the entire study period (2018–2022). Some listening stations had known gaps in acoustic data collection related to limited field operations, equipment failure, only seasonal recording periods, and/or data quality issues (Fig. S1).

### Sanctuary-wide comparisons of vessel traffic by size

Vessel composition, represented by vessel size category from AIS data, varied across the sanctuary system, as well as within some sanctuaries (Fig. 1D). Some listening stations were dominated by large vessels (e.g., Fig. 1D MB03), some a mix of vessel sizes (e.g., Fig. 1D HI03), and others, had mainly small vessels (e.g., Fig. 1D GR01).

Vessel size categories from AIS provided a proxy for the type of nearby vessel traffic and operating behavior. Listening stations with a higher proportion of small vessels (Fig. 1D) likely represent locally operated vessels on schedules tied to weather and seasonal factors (e.g., fishing seasons). Stations with a higher proportion of medium-sized vessels (e.g., Fig. 1D CI02, HI01, MB01) likely had vessels operating on regional scales (e.g., commercial whale watching and fishing vessels) and using varied routes. Stations with a higher proportion of large vessels transiting nearby (e.g., Fig. 1D OC02, CI05, SB03) were situated near designated shipping routes with regular passages of large commercial vessels (Fig. 1C, e.g., container ships) within the 10 km buffer.

### Categorizing listening stations according to vessel noise

**Fig. 2:**
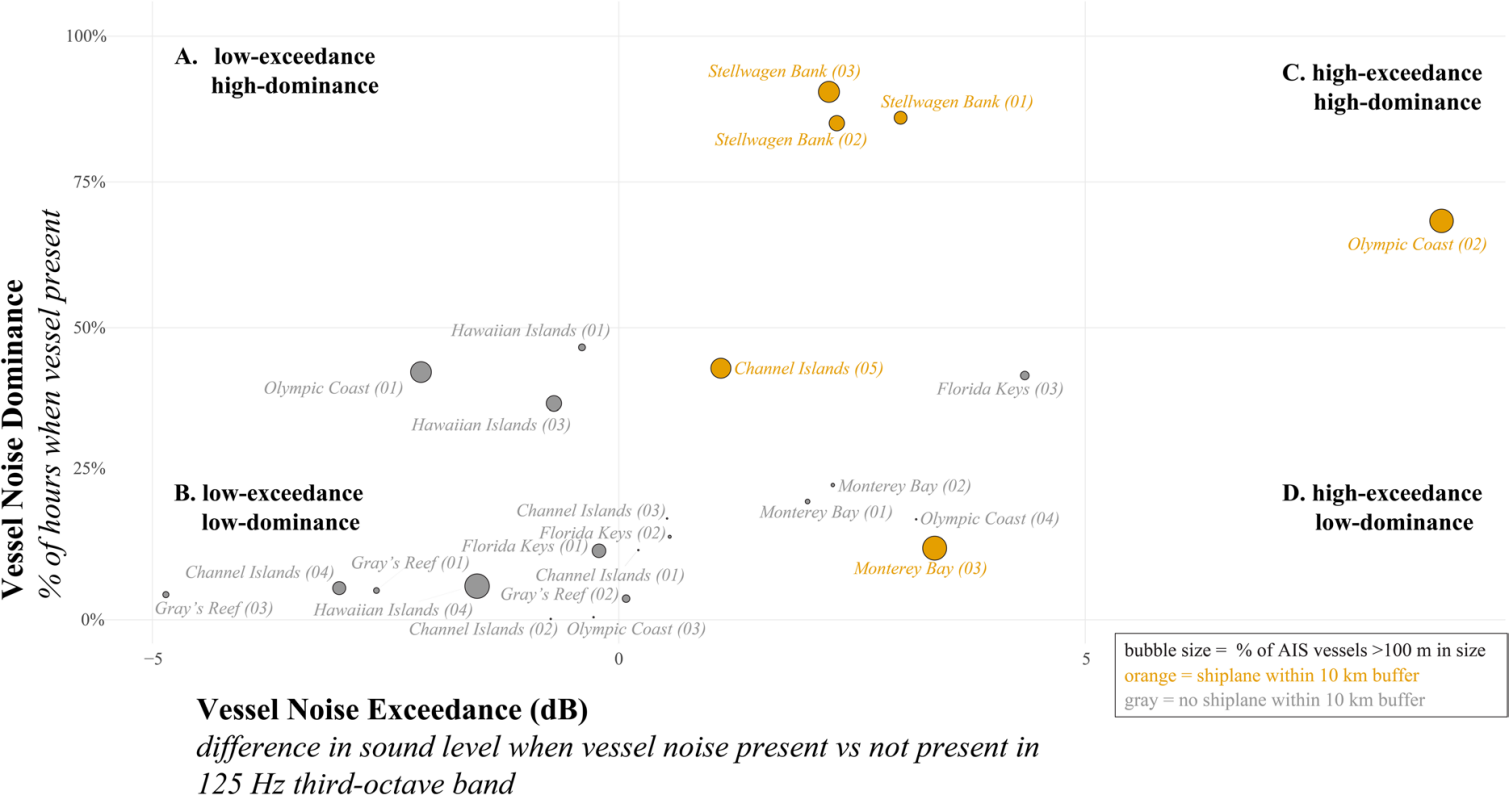
Vessel noise categories across 24 listening stations, one site (PM08) did not have vessel noise detections. A single month in 2019 is shown for each listening station (see Fig. S1 for specific month). Vessel noise exceedance (dB) (x-axis) was calculated as the difference in hourly median sound level in the 125 Hz one-third octave band (decibels) when a vessel was acoustically detected vs not detected across all hours in a month; negative values occur when on average the acoustic environments were higher when no vessels were present. Vessel noise dominance (y-axis) was calculated as the percentage of the hours (in a month) vessel noise was detected in the soundscape. Four general categories of vessel noise are shown based on dominance and exceedance: (**A**) upper left includes listening stations with high vessel dominance but low-exceedance; (**B**) listening stations in upper right had both high-exceedance and dominance; (**C**) lower left shows listening stations with minimal vessel presence (low-exceedance and dominance); (**D**) lower right represents listening stations with high-exceedance but vessels not detected as often. The size of the bubbles is % of AIS vessels greater than 100 m in length (large size category, see Fig. 1); orange bubbles indicate a shipping lane was within the 10 km buffer, and gray bubbles indicate no shipping lane within the 10 km buffer around a listening station

We used multiple acoustically derived metrics to describe the variation in vessel noise across the NMS system and categorized listening stations into four general categories: high-dominance, high-exceedance; high-dominance, low-exceedance; low-dominance, high-exceedance; low-dominance, low-exceedance (Table 1, Fig. 2). The vessel noise dominance metric describes the amount of time that vessel noise is present in the soundscape. The vessel noise exceedance metric quantifies the noise that is added to soundscape when vessel is acoustically detected.

Most listening stations within the high-exceedance and high-dominance quadrant had a designated commercial shipping lane within the 10 km buffer (e.g. Fig. 2 OC02, SB01), resulting in continuously present vessel traffic (high-dominance) as well as larger commercial vessels transiting nearby with higher low-frequency source levels (high-exceedance). Previous studies showed that larger commercial ships have higher source levels (MacGillivray et al., 2019; MacGillivray & de Jong, 2021). Listening stations within the low-exceedance yet high-dominance category (e.g., Fig. 2A HI01) indicated there were other low-frequency sounds contributing to the soundscape, and vessel noise detections were lower than these other sources in the soundscape. In some cases, the difference may be small because vessels were transiting further away from the listening station (no designated shipping lane nearby), resulting in low sound levels during vessel detection periods. Listening stations with high-exceedance had low ambient sound levels when vessels were not acoustically detected. Many of these stations had a low percentage of large vessels present (e.g., Fig. 2 MB01) indicating that vessel traffic was mainly from smaller vessels, operating at regional and local scales. The 11 stations in the low-exceedance and low-dominance quadrant had low vessel noise presence during the analyzed month in 2019.

### Seasonal and daily variation in vessel noise

**Fig. 3:**
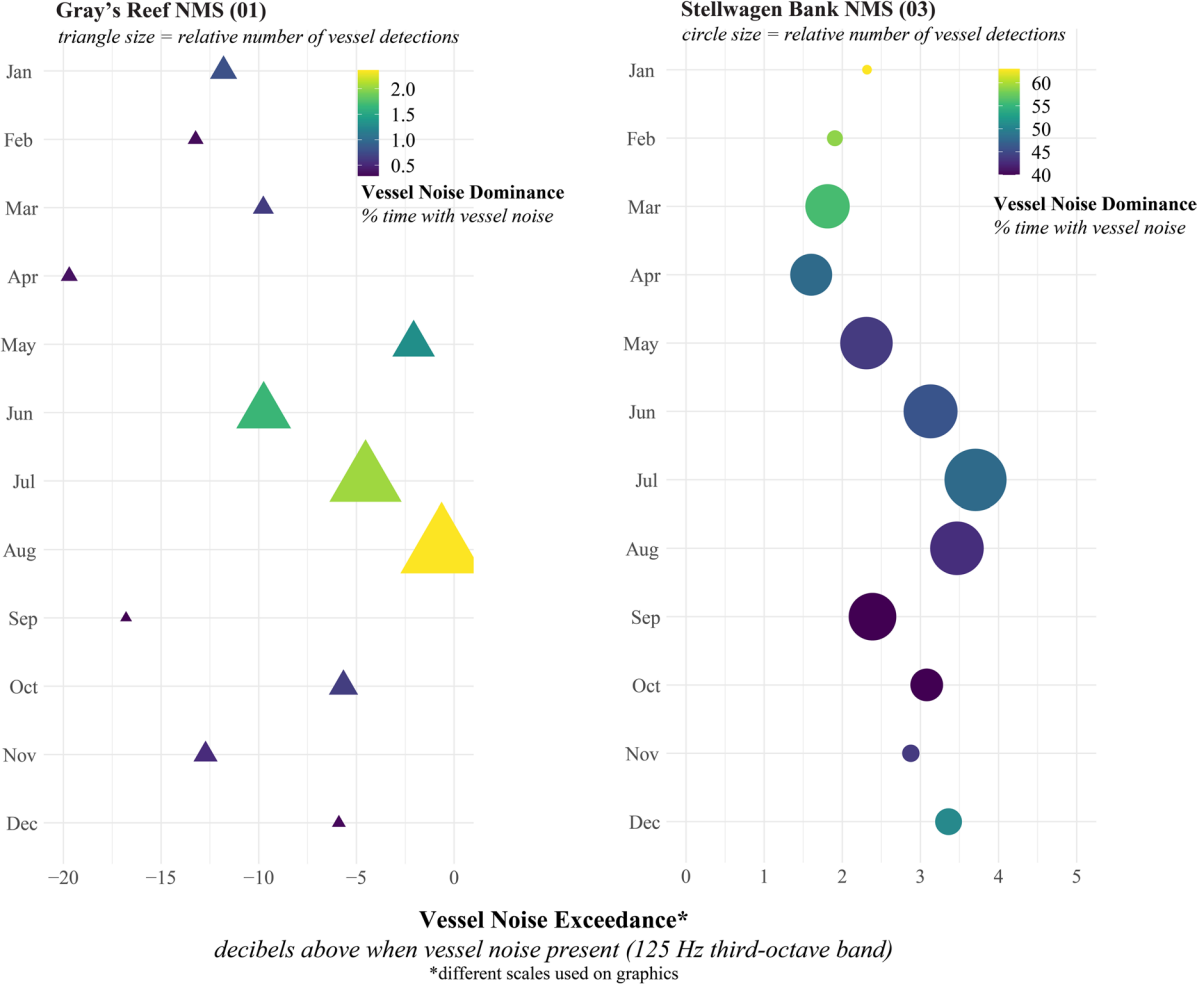
For each month in 2019 at two listening stations, noise exceedance (x-axis, calculated as the difference in maximum low-frequency sound level at 125 Hz one-third octave band when vessel was detected compared to closest non-vessel period) and vessel noise dominance (color, percent of time with vessel noise present) are shown. Note different noise dominance scales for each site, represented by shapes. Relative number of vessel acoustic detections for each site (shape size) also varied by month, with highest numbers in summer months at both listening stations. At SB03 total number of monthly vessel acoustic detections ranged from 333 (Feb) to 583 (Jul) and at GR01 total number of monthly vessel detections ranged from 9 (Sep) to 78 (Aug)

Vessel traffic and other contributions to the soundscape were dynamic throughout the year, resulting in seasonal shifts in vessel noise (Fig. 3). For two listening stations in different vessel noise categories (Stellwagen Bank NMS and Grey’s Reef NMS), we examined how exceedance and dominance metrics shifted across an entire year (Fig. 3). Overall, both listening stations remained in their respective quadrants of vessel noise presence: Stellwagen Bank NMS (03) had high-exceedance (1–4 dB) and high-dominance (40–60%) and Gray’s Reef NMS (01) had low-exceedance (−15 to −2 dB) and low-dominance (0–2%). At GR01, low-frequency sound levels during vessel detection periods were lower than non-vessel periods, with the most deviation in April and September, suggesting other sources (e.g., fish, T. Rowell, per. comm.) increase low-frequency sound levels when vessel noise was not detected in the soundscape. Vessel noise dominance or the percentage of time vessel noise was detected in the soundscape increased in summer months (~2% of time for GR01). June 2019, for example, had higher vessel noise dominance (2%), yet noise exceedance remained low (−10 dB). Vessel operational behavior (speed and distance to the stations) and presence of biological sounds influenced this metric of noise exceedance.

Although chronically influenced by vessel noise throughout the year, seasonal variation in exceedance at SB03 was likely related to difference in wind-driven noise, where winter months (November–February 2019) showed lower vessel noise exceedance due to higher wind-driven background noise conditions compared to summer (June–August 2019) (Fig. 3). Mandatory vessel speed reduction in place to protect whales from ship strike risk in March–April 2019 resulted in lower vessel noise exceedance levels, as is further explored in the next section. Interestingly, months with higher noise dominance (January–February 2019) at SB03 had a relatively lower number of vessel detection periods (Fig. 3), indicating that durations of vessel noise detections were longer in these months. Summer 2019 months in Stellwagen Bank NMS had more vessel types present, resulting in overlapping vessel passages and leading to fewer unique detection periods, yet higher vessel noise exceedance (Fig. 3).

**Fig. 4:**
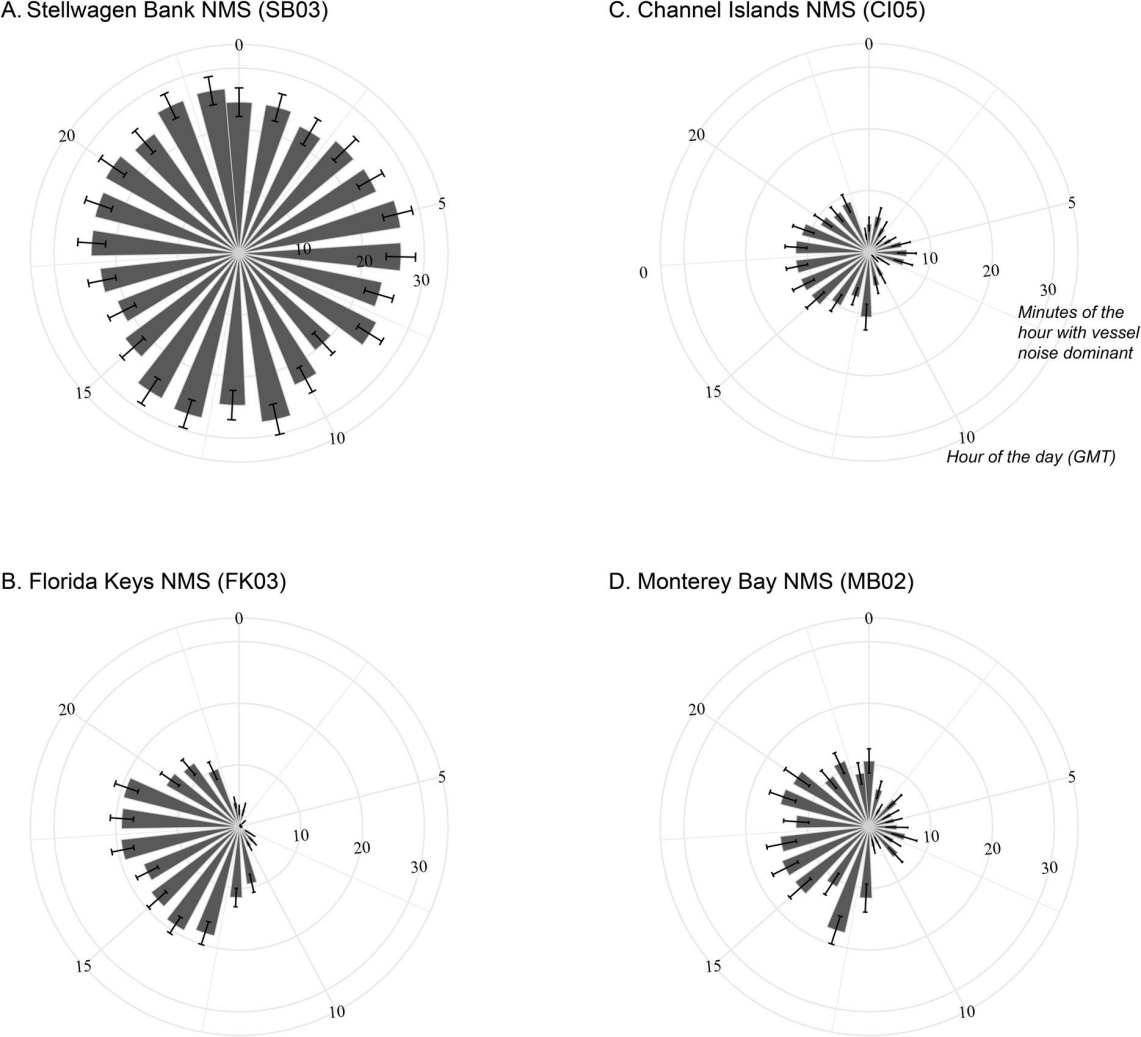
Daily patterns in vessel noise dominance. Listening stations (**A**) SB03, (**B**) FK03, (**C**) CI05, and (**D**) MB02 all occurred in the *high-exceedance* category (Fig. 2), yet different daily patterns emerge when summarized as average minutes of the hour dominated by vessel noise. Standard error for each hour within a single month in 2019 (see Figure S1)

Daily distributions of vessel noise dominance and exceedance were examined to assess diurnal patterns in human use and biological presence in soundscapes (Fig. 4), as well as evaluate these acoustic metrics on a finer temporal scale. At four listening stations in the high vessel noise exceedance category (Fig. 2), the hours of the day affected by vessel noise varied. At SB03, across all hours of the day, 30 min of each hour were dominated by vessel noise (Fig. 4A), in contrast to the three other listening stations where vessel noise occurred mainly in certain hours of the day. FK03 showed that 10–20 min of each hour had vessel noise, but only during daytime hours (Fig. 4B). Two listening stations (CI05 and MB02) showed even lower minutes of hours with vessel noise (10–15 min), occurring at specific periods in the day (Fig. 4C, D).

### Vessel slowdowns and influence on vessel noise exceedance

**Fig. 5:**
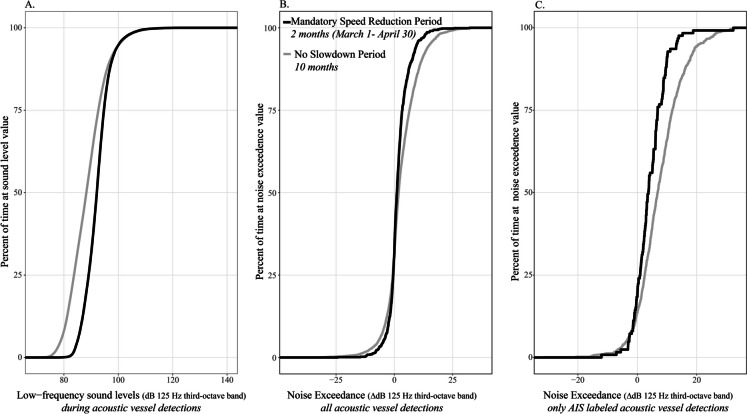
Comparisons of different vessel noise metrics between mandatory vessel speed reduction period and no vessel slowdown period in Stellwagen Bank NMS. All months in 2019 were analyzed. Empirical Cumulative Distributions, representing the percent of time (1-min samples) a specific x-axis variable occurs, are shown for (**A**) 1-min low-frequency sound levels (125 Hz third-octave band), only during acoustic vessel detections, (**B**) noise exceedance for all acoustic vessel detections within the two time periods, and (**C**) noise exceedance for acoustic vessel detection with known AIS vessel transiting within 10 km of the listening station. Noise exceedance quantifies the difference between maximum sound level during an acoustic vessel detection period compared to nearest non-vessel period

Using the data collected over the entire year in 2019 at SB03 (Fig. S1), we examined how combining vessel noise exceedance with AIS data showed a co-benefit of noise reduction (Fig. 5) during a collision-risk reduction focused vessel speed reduction period. The mandatory slowdown was 2 months (April–May) and the rest of the year did not have a vessel slowdown requirement. We compared the distributions of vessel noise metrics in each period, “mandatory speed reduction period” and “no slowdown period,” as a way to compare the percentage of the time at different noise values. We first plotted low-frequency sound level data (125 Hz third-octave band) when vessels were acoustically detected in each period; the distribution of low-frequency sound levels (or percent of the time at different sound levels) did not show a shift to more time at lower sound levels during the slowdown months (Fig. 5A); in fact, vessel detection periods were 3–5 dB higher during the vessel slowdown period. However, when we calculated vessel noise exceedance in the two periods (slowdown and non-slowdown), vessel noise exceedance distributions (% of the time) shifted to lower exceedance values at higher percent of the time during the slowdown period compared to the non-vessel slowdown period (Fig. 5B). For example, 75% of the time noise exceedance was at or below 2 dB for slowdown period, whereas for no slowdown period 75% of the time noise exceedance was at or below 4 dB. We then integrated AIS vessel presence within 10 km of the listening station to only look at vessel noise exceedance when a known AIS vessel was present (Fig. 5C). In this case, vessel noise exceedance was less during the slowdown period for a higher percentage of the time sampled, suggesting a noise reduction benefit. This addition of AIS was valuable in this case because only larger AIS vessels were mandated to slow down, and larger commercial vessels tended to travel at higher speeds and generate higher noise levels (MacGillivray et al., 2019). Therefore, these ships would experience the greatest reduction in noise during the slowdown, resulting in the greatest changes in vessel noise exceedance.

### Shifts in vessel patterns and influence on noise dominance

**Fig. 6:**
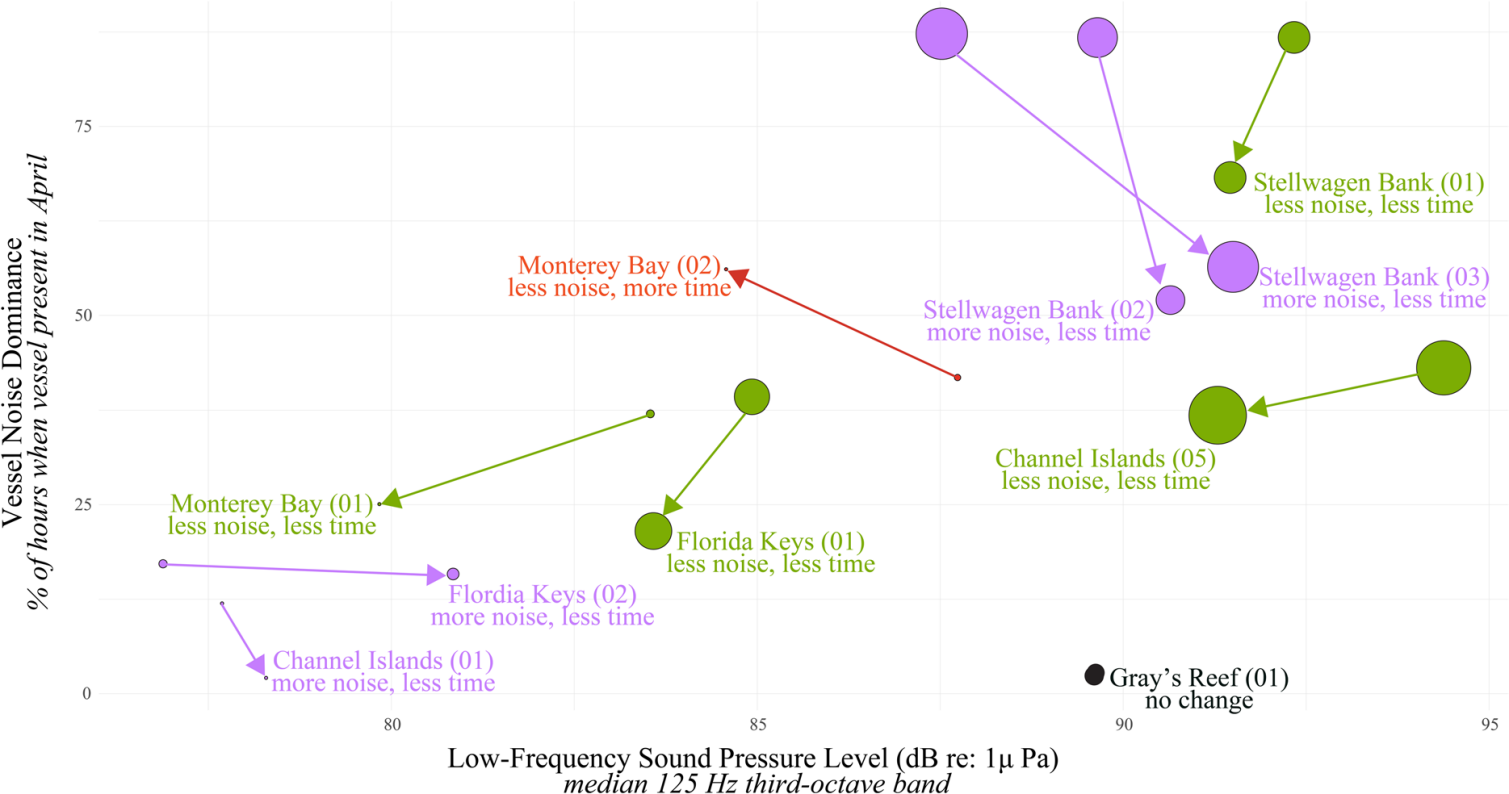
Vessel noise changes related to early COVID-19 pandemic shutdowns. A comparison of conditions in April 2019 compared to April 2020—arrow direction points from 2019 to 2020. The x-axis shows sound pressure level (not vessel noise exceedance) because a change in noise exceedance is not expected assuming vessels did not change routes in relation to the site. Further, we were interested in how the likely reduction in vessel traffic reduced median low-frequency sound levels at each site. Colors represent categories of how vessel noise presence changed: **green**=reduction in both, **purple**=reduction in noise dominance and increase in sound level, **red**=increase in noise dominance and reduction in sound level, **black**=no change. Bubble size is the proportion of vessel traffic in the month that were large vessels and size change for a given site indicates a shift in traffic composition

Early in the COVID-19 pandemic, human activities shifted in coastal waters. To understand how these shifts changed underwater vessel noise, we compared nine listening stations with data collected in April 2019 and 2020 (Fig. 6). We focused on vessel noise dominance (% of the time vessel noise present) for this analysis because this metric was more sensitive to reductions in vessel activity. We included metrics from the AIS data (percent of vessel in large category, <100 m) to help interpret the changes. Changes in conditions before vs. early in the COVID-19 pandemic were not consistent across listening stations in the same sanctuary or among listening stations within the same vessel noise categories (Fig. 2). This indicated both local and regional differences in pandemic-linked drivers influencing vessel noise metrics. Reduction in vessel noise dominance during the early pandemic was observed at seven of the nine listening stations examined; however, only four of those stations experienced a corresponding reduction in low-frequency sound levels expected due to fewer vessels transiting nearby (Fig. 6). MB01 showed the most dramatic reduction in low-frequency sound levels during the early pandemic (Fig. 6), related to reductions in regional vessel traffic, especially the larger vessels (Ryan et al., 2021). Another listening station within the same sanctuary showed an increase in vessel noise dominance (more time with vessel noise) but a decrease in low-frequency sound levels (Fig. 6 MB02), likely related to reductions in certain vessel in the area, (e.g., whale watching). At four listening stations, a reduction in vessel noise dominance was observed, but low-frequency sound levels increased, which may indicate more influence from other sound sources in the 2020 soundscape (e.g., wind, biological). At stations with very low vessel activity (Fig. 6 GR01), no change during the early pandemic was observed.

## Discussion

Standardized monitoring of marine vessel activity and related underwater radiated noise across a network of protected areas, the U.S. National Marine Sanctuary system, created opportunities to compare a spectrum of vessel noise conditions and help guide strategies to mitigate impacts. By using information from a robust, integrated, and multi-dimensional monitoring program, the SanctSound project, standardized acoustic data, and contextual variables were readily available to provide insight on the complex dynamics of vessel activity across wide-ranging network of marine protected areas (MPAs). The potential application of these methods and metrics is diverse, including condition assessments, evaluating management actions, prioritizing funding, and leveraging resources. Decisions regarding the application are informed by the management context and beyond the scope of this paper.

We categorized listening stations based on both the amount of time that vessel noise is present in the soundscape (dominance) and the level of noise added by vessels when they are present (exceedance). While we did not identify specific thresholds in this study, practitioners could appropriately set thresholds to correspond with different management objectives (Buxton et al., 2019; Hatch & Fristrup, 2009; Joint Research Centre (European Commission) et al., 2023), furthering the application of these metrics. The metrics we used to describe the spectrum of conditions across sites (Fig. 2), seasons (Fig. 3), and hours (Fig. 4) showcase one type of comprehensive assessment of vessel noise across a diverse network of MPAs. Combining this knowledge with vulnerable resources (e.g., endangered species) and community needs can help inform efficient and effective strategies for reducing and/or mitigating vessel noise within the U.S. National Marine Sanctuary System and other MPA networks. These results could also be leveraged for understanding other vessel-related threats to sanctuary resources or assessing value based on human-use patterns across MPAs.

AIS vessel traffic varied regionally (Fig. 1D)—influenced by local vessel use patterns and global commercial trade routes. Threats from vessels, including underwater radiated noise, are well-documented with consequences to marine species and ecosystems (Erbe et al., 2019; Weilgart, 2018). A requisite to effective MPA vessel noise monitoring is indicator metrics that capture a variety of conditions. To develop an approach to describe vessel noise across networked MPAs, we integrated metrics from multiple monitoring technologies to produce scalable indicators of conditions at 25 listening stations within seven U.S. marine sanctuaries and one marine national monument.

Within the NMS system context, we defined categories of dominance and exceedance to group listening stations with similar vessel noise conditions. Soundscapes with comparatively high-exceedance and high-dominance vessel noise and with a commercial shipping lane nearby had AIS traffic patterns composed of mainly large ocean-going commercial vessels (Fig. 2C). Sanctuaries with a lower proportion of large AIS vessels present and low vessel noise dominance, but with high influence of vessel noise when vessels are present (high-exceedance) likely require finer temporal assessment to understand when the high-exceedance occurred (Fig. 4). Soundscapes in the low-exceedance yet high-dominance category (Fig. 2A) suggested the presence of mostly smaller non-AIS vessel traffic with lower noise emissions, complimenting the insight gained from AIS technology. In some cases, these conditions (the low-exceedance yet high-dominance) indicate a high non-vessel background noise condition, highlighting the value of multiple metrics to describe vessel noise at a given location. Listening stations categorized as low-exceedance and low-dominance represented locations with currently low impacts from vessels and the potential opportunity to maintain low vessel noise conditions within these MPAs. For each category of vessel noise impact, integrating information on the species inhabiting these protected waters and societal context can help direct opportunities for reducing vessel noise, such as establishing and maintaining noise-free periods, enhancing port efficiency, engaging with regional and international vessel quieting initiatives, and leveraging noise reduction co-benefits from management actions for reducing other vessel-related impacts.

Each listening station monitored in this study represents a specific location in an MPA, and collective sounds recorded come from a variable area around the station. Vessel noise, for example, can propagate to each listening station at different distances—depending on vessel types and operational conditions in the area, the topography and composition of the seafloor, and temperature and salinity of the water. Careful selection of monitoring sites and understanding the area they represent is key for interpreting the results beyond a single listening station location. Estimating the area over which vessels (and other sounds) are contributing to a listening station provides a spatial estimate for interpretation. For example, the larger SanctSound project estimated ranges for specific vessel types, and comparisons (e.g., seasonal) can be examined using the project data portal (Margolina, 2020) (https://sanctsound.portal.axds.co/). While the analyses presented in this study did not incorporate the spatial range of vessel detection, doing so would provide a spatial estimate over which management actions may be effective, an important next step.

Targeted strategies to reduce vessel noise in national marine sanctuaries include initiatives to design and operate vessels more quietly (Boyd et al., 2011; Findlay et al., 2023). Where traffic lanes transiting sanctuaries are accessing nearby ports, complementary soundscape improvement strategies can be explored as co-benefits to efficiency measures for port operations. For example, optimized schedules that concentrate the transfer of goods to certain periods of the day (Fig. 4), can result in reliable quieter periods and reduce vessel noise overlap and interference with biological sound production (Fournet et al., 2018; Haver et al., 2023). Further, fine-scale temporal evaluation of vessel noise can reveal opportunities for management approaches to achieve species-specific conservation goals. For example, although vessel noise dominance in Stellwagen Bank NMS is chronically high, exceedance is highest in summer months (Fig. 3) when several populations of large whales are feeding.

Another requisite to effective MPA vessel noise monitoring is indicator metrics that are sensitive to shifts in vessel dynamics. We evaluated sensitivity of the vessel noise metrics to known changes in vessel use patterns. First, we examined how early COVID-19 pandemic shifts in human activity patterns changed vessel noise dominance, expecting an overall reduction from reduced marine commercial trade and tourist activity. Numerous studies have documented these changes, including the reduction in marine vessel traffic (Depellegrin et al., 2020; Huveneers et al., 2021; March et al., 2021) and associated noise (Gabriele et al., 2021; Ryan et al., 2021). Listening stations in this study that monitored for vessel noise before and during the pandemic showed a change in vessel noise dominance, with the exception of GR01 which showed no change (Fig. 6). Most stations analyzed in this study (8 of 10) showed a reduction in noise dominance (the time vessel noise was present in the soundscape), representing a general reduction in vessel activity across sanctuaries. For listening stations that showed an increase in noise dominance (Fig. 6 MB02), interesting shifts in vessel use patterns in 2020 likely occurred. For example, AIS data revealed that during the early COVID-19 pandemic there were fewer large AIS vessels but smaller AIS fishing vessels came closer to the listening station at MB02 (https://vessel-traffic-esrioceans.hub.arcgis.com/). The vessel noise exceedance metric was less useful, especially at listening stations with commercial shipping lanes nearby. Instead, low-frequency sound levels, a commonly used measure of marine vessel activity in high commercial shipping areas (Haver et al., 2021), was examined for any reductions in sound levels. The low-frequency sound levels showed minor reductions, and in some cases increases, indicating vessel noise dominance proved to be a more sensitive metric to changes in vessel activity related to the early COVID-19 pandemic. Increased low-frequency sound levels during the early COVID-19 pandemic observed at some listening stations (4 of 10) may have been driven by non-vessel sources (e.g., biological, wind). Further analysis would be necessary to examine whether increases in biological activity correlated with reductions in vessel activity.

To understand the sensitivity of the vessel exceedance metric, we examined exceedance during a mandatory vessel speed reduction management action, expecting a reduction due to lower noise output from slower vessels (Findlay et al., 2023; MacGillivray et al., 2019; McKenna et al., 2013; ZoBell et al., 2021). In line with the expectation, a reduction in vessel noise exceedance was measured (Fig. 5), and the addition of AIS labeling of the data was valuable to interpret these changes. Typically, only larger AIS vessels are mandated to slow down, and these vessels tend to travel at higher speeds and generate higher noise levels. These AIS ships, therefore, experienced the greatest reduction in noise during the slowdown, resulting in the greatest changes in vessel noise exceedance, as seen in Fig. 5C. Vessel speed reduction also mitigates multiple social and environmental impacts of maritime transportation, namely reduction in greenhouse gas emission, improvement in air quality, reduction in lethal collisions with wildlife and non-living objects, and reduction in the spread of invasive species (Sèbe et al., 2022). Consideration of feasibility, compliance, and navigational safety is key to successful implementation of vessel slowdown initiatives (Haren, 2007), and a variety of slowdown programs have demonstrated the benefits to marine ecosystems and species (Breeze et al., 2022; Lo et al., 2022; Williams et al., 2019; ZoBell et al., 2021).

The value of using vessel noise indicators to describe MPA soundscapes and inform management depends on longevity and consistency of the monitoring and analysis effort. Monitoring the status and trends of natural resources and human uses often relies on indices derived from remote sensing technologies, like acoustic monitoring, to provide simplified but representative descriptions of conditions over broad spatial and temporal scales (El Mahrad et al., 2020). For example, this study underscores that continued and strategic passive acoustic monitoring could allow U.S. national marine sanctuary managers to track vessel noise indicators over time and at spatial scales relevant to their management priorities. Standardized, accessible, and cost-effective data processing routines are also necessary and key to not only this study but also for successful implementation of future vessel noise monitoring programs (Wall et al., 2021). Further, with a robust, integrated, and comprehensive vessel noise monitoring program in place, when management strategies are implemented, either MPA-specific or at greater scales that influence vessel operations within and around the protected area, the assessment of effectiveness or co-benefits is possible and scalable across the network.

## Conclusion

By leveraging a multi-year cross agency collaborative environmental monitoring effort, we comprehensively assessed a major concern in coastal marine ecosystems: the impacts of vessel-generated noise in marine protected areas. Standardized monitoring of marine vessel activity and related underwater radiated noise across a network of protected areas within the U.S. National Marine Sanctuary system provided valuable insights on the variety of existing conditions that can help guide strategies to mitigate noise pollution impacts. We further demonstrated that our approach and metrics are robust and detect changes in conditions related to both external drivers (COVID-19 pandemic) and internal management actions (mandatory vessel slowdowns). Collectively, these results illustrate scalable opportunities for monitoring and reducing vessel noise across a variety of conditions within marine protected areas.

### Supplementary Information

Below is the link to the electronic supplementary material.

Supplementary file1 (DOCX 318 KB)

## Data Availability

The acoustic data are available through the Sanctuary Soundscape data portal: https://sanctsound.portal.axds.co/) and raw audio files are available through National Center for Environmental Information (https://www.ncei.noaa.gov/products/passive-acoustic-data). All code to process the data is shared via GitHub repository (https://github.com/mfmckenna/MM_SanctSound_VesselNoise)
