# Supplementary material for: Understanding vessel noise across a network of marine protected areas
Source: Environ Monit Assess. 2024 Mar 15;196(4):369. doi: 10.1007/s10661-024-12497-2 (PMC10942938; doi:10.1007/s10661-024-12497-2)
Supplement: Supplementary file 1 — Supplementary file1 (DOCX 318 KB) [file 10661_2024_12497_MOESM1_ESM.docx]

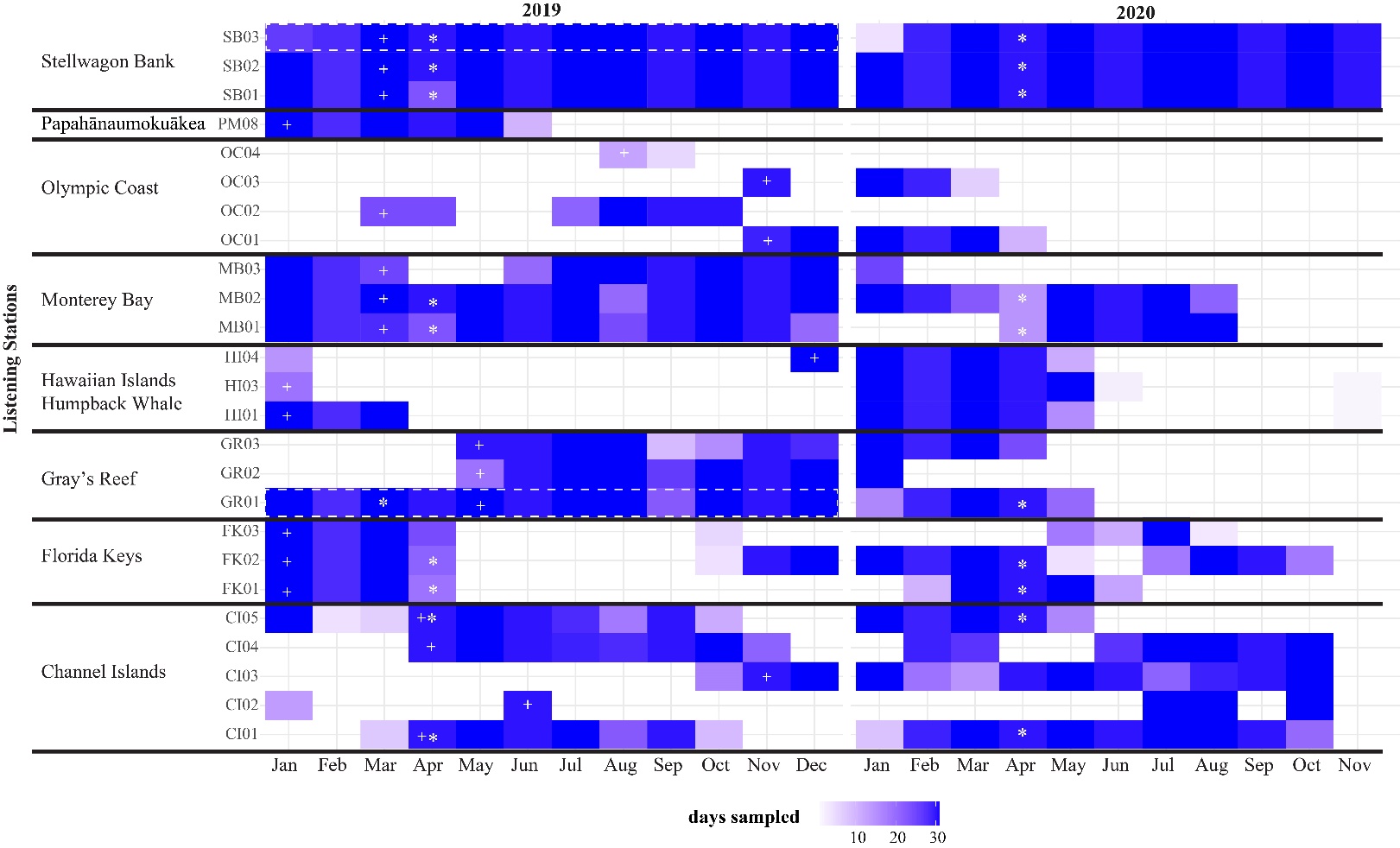


**Figure S1:** Passive acoustic monitoring data from each listening station used in the vessel noise analysis (n=25 locations). Plus signs in boxes (+) indicate station-months used in all the 2019 analyses (Figures 2-3). Listening stations used in seasonal comparison are marked with white dashed boxes (Figure 4). Months used in the early COVID-19 pandemic comparison are marked with an asterisk (*) (Figure 5). Shading represents days with data for a given month that was available at the time of analysis (https://sanctsound.portal.axds.co/). There was missing data (blank months) because of equipment loss, equipment failure, seasonal recording schedule, or data quality issues.

Table S1: Vessel Noise Detector settings

| **Sanctuary** | **Summary of vessel noise detector settings. Detector requires MATLAB 2016b or newer and signal processing and statistics toolboxes** [**https://github.com/MarineBioAcousticsRC/Triton/tree/master/Remoras/Ship-Detector**](https://github.com/MarineBioAcousticsRC/Triton/tree/master/Remoras/Ship-Detector) |
| --- | --- |
| Stellwagen Bank National Marine Sanctuary | Low band limit (Hz): 50-3,000, Medium band limit:3,000-10,000, High band limit: 10,000-17,000, Close passage duration threshold (s): 25, Distant passage duration threshold (s): 30, Received level threshold (%): 0.03, Time between passages (h): 0.1, Buffer time (min): 4, Window Size (h): 2, Sliding Time Overlapping Windows (h) 0.5. |
| Grays Reef National Marine Sanctuary | Low band limit (Hz): 100-2,000, Medium band limit: 2,000-3,000, High band limit: 3,000-4,000, Close passage duration threshold (s): 40, Distant passage duration threshold (s):45, Received level threshold (%): 0.03, Time between passages (h): 0.1, Buffer time (min): 4, Window Size (h): 1, Sliding Time Overlapping Windows (h) 0.5. |
| Florida Keys National Marine Sanctuary | Low band limit (Hz):100-3,000, Medium band limit: 3,000-4,000, High band limit: 4,000-5,000, Close passage duration threshold (s): 20, Distant passage duration threshold (s):25, Received level threshold (%): 0.01, Time between passages (h): 0.01, Buffer time (min): 4, Window Size (h): 1, Sliding Time Overlapping Windows (h) 0.5. |
| Channel Islands National Marine Sanctuary | Low Band=100-5,000 Hz, Medium Band=5,000-10,000 Hz, High Band=10,000–48,000 Hz, Close Passage Duration Threshold: 50 s, Distant Passage Duration Threshold: 150 s, Relative Threshold Level = 0.05%, Time Between Passages = 0.01 hr, Buffer Time (min): 5, Window Size (h): 2, Sliding Time Overlapping Windows (h) = 0.5 |
| Monterey Bay National Marine Sanctuary | Low Band=100-5,000 Hz, Medium Band=5,000-10,000 Hz, High Band=10,000–48,000 Hz, Close Passage Duration Threshold: 50 s, Distant Passage Duration Threshold: 150 s, Relative Threshold Level = 0.05%, Time Between Passages = 0.01 hr, Buffer Time (min): 5, Window Size (h): 2, Sliding Time Overlapping Windows (h) = 0.5 |
| Olympic Coast National Marine Sanctuary | Low Band=100-5,000 Hz, Medium Band=5,000-10,000 Hz, High Band=10,000–48,000 Hz, Close Passage Duration Threshold: 50 s, Distant Passage Duration Threshold: 150 s, Relative Threshold Level = 0.05%, Time Between Passages = 0.01 hr, Buffer Time (min): 5, Window Size (h): 2, Sliding Time Overlapping Windows (h) = 0.5 |
| Hawaiian Islands Humpback Whale Marine Sanctuary HI01 | Low band limit (Hz): 100-3,000, Medium band limit: 3,000-4,000, High band limit: 4,000-7,500, Close passage duration threshold (s): 20, Distant passage duration threshold (s): 50, Received level threshold (%): 0.01, Time between passages (h): 0.01, Buffer time (min): 1, Window Size (h): 1, Sliding Time Overlapping Windows (h) 0.5. |
| Hawaiian Islands Humpback Whale Marine Sanctuary HI03 | Low band limit (Hz): 100-3,000, Medium band limit: 3,000-5,000, High band limit: 5,000-7,5000, Close passage duration threshold (s): 40, Distant passage duration threshold (s): 45, Received level threshold (%): 0.01, Time between passages (h): 0.1, Buffer time (min): 0.083, Window Size (h): 1, Sliding Time Overlapping Windows (h) 0.05. |
| Hawaiian Islands Humpback Whale Marine Sanctuary HI04 | Low band limit (Hz): 100-5,000, Medium band limit: 5,000-7,000, High band limit: 7,000-15,000, Close passage duration threshold (s): 40, Distant passage duration threshold (s): 45, Received level threshold (%): 0.1, Time between passages (h): 0.1, Buffer time (min): 5, Window Size (h): 1, Sliding Time Overlapping Windows (h) 0.5. |
| Hawaiian Islands Humpback Whale Marine Sanctuary HI05 | Low band limit (Hz): 100-3,000, Medium band limit: 3,000-4,000, High band limit: 4,000-7,500, Close passage duration threshold (s): 20, Distant passage duration threshold (s): 50, Received level threshold (%): 0.01, Time between passages (h): 0.01, Buffer time (min): 1, Window Size (h): 1, Sliding Time Overlapping Windows (h) 0.5. |
| Hawaiian Islands Humpback Whale Marine Sanctuary HI06 | Low band limit (Hz): 100-3,000, Medium band limit: 3,000-7,500, High band limit: 7,500-15,000, Close passage duration threshold (s) 40: , Distant passage duration threshold (s): 45, Received level threshold (%): 0.05, Time between passages (h): 0.1, Buffer time (min): 1, Window Size (h): 1, Sliding Time Overlapping Windows (h) 0.3. |
| Papahānaumokuākea Marine National Monument  PM08 | Low band limit (Hz): 100-1,000, Medium band limit: 1,000-3,000, High band limit: 3,000-5,000, Close passage duration threshold (s): 100, Distant passage duration threshold (s): 200, Received level threshold (%): 0.1, Time between passages (h): 0.25, Buffer time (min): 5, Window Size (h): 2, Sliding Time Overlapping Windows (h) 1. |

| **Table S2: Additional vessel noise metrics** | | |
| --- | --- | --- |
| **Description** | **Figure** | **Interpretation** |
| A sum of the durations of all AIS vessel tracks within a 10 km buffer. Metric is calculated as an average of all days in a month. | Not shown | Higher values indicate vessels are nearby for a higher percentage of the time and likely overlapping vessels present within the 10 km buffer. |
| Total of all unique vessels equipped with AIS within a 10 km buffer around a listening station. Metric is calculated as both an average and standard deviation of each day of a month. | Not shown | Higher values indicate listening stations with predictable routes nearby with a diversity of transiting vessels (e.g., commercial shipping routes). Higher variation indicates vessel activity related to day of the week, weather, or time of the month. |
| Total of all vessel dominant periods in the acoustic data. Metric is calculated as both an average and standard deviation of each day of a month. | Not shown | Higher values indicate listening stations with predictable routes nearby either by a mix of vessels or routine vessel traffic. Higher variation indicates vessel activity related to day of the week, weather, or time of the month. |
